# SaCas9-Mediated *CrtYB* Disruption Coupled with Heterologous α-Bisabolol Production in *Rhodotorula mucilaginosa* JY1105

**DOI:** 10.3390/microorganisms14102205

**Published:** 2026-10-01

**Authors:** Wenyi Sun, Haoyang Liu, Yaqi Dong, Haoyu Li, Yanan Wang, Qian Cai

**Affiliations:** 1 School of Pharmacy, Liaoning University of Traditional Chinese Medicine, Dalian 116600, China; 2 State Key Laboratory Breeding Base of Dao-di Herbs, National Resource Center for Chinese Materia Medica, China Academy of Chinese Medical Sciences, Beijing 100700, China

**Keywords:** *Rhodotorula mucilaginosa*, SaCas9, CRISPR/Cas9, *CrtYB*, α-bisabolol, terpene biosynthesis

## Abstract

*Rhodotorula mucilaginosa* is a promising host for terpene biosynthesis, but its rational engineering remains constrained by limited genome-editing tools. Here, we developed a *Staphylococcus aureus* Cas9 (SaCas9) workflow in *R. mucilaginosa* JY1105 for *CrtYB* disruption and demonstrated heterologous α-bisabolol production. Five sgRNAs targeted CrtYB, encoding bifunctional phytoene synthase/lycopene cyclase; its disruption provided visible depigmentation for preliminary screening. Among 25 sequenced transformants per guide, CrtYB3 and CrtYB5 yielded 10 (40%) and 4 (16%) white colonies, respectively; each white colony carried an on-target deletion of 11–26 bp, whereas all red colonies retained the wild-type target sequence. No white colonies or target-site deletions were detected for CrtYB1, CrtYB2, or CrtYB4. A codon-optimized chamomile TPS1 cassette encoding α-bisabolol synthase was integrated by random integration into wild-type JY1105 and the edited R1-ΔCrtYB derivative. The selected highest producers produced 37.52 ± 3.09 and 41.45 ± 2.08 mg L^−1^ α-bisabolol, respectively. To our knowledge, this is the first report of heterologous α-bisabolol production in *R. mucilaginosa*. This study demonstrates the potential of *R. mucilaginosa* as an editable chassis for heterologous sesquiterpene biosynthesis and provides a foundation for further development of SaCas9-assisted metabolic engineering in this non-conventional yeast.

## 1. Introduction

Terpenoids constitute a structurally diverse class of natural products with broad applications in food, fragrances, cosmetics, pharmaceuticals, and renewable chemicals. Compared with direct extraction from plants, microbial cell factories provide a more controllable and potentially sustainable route for terpenoid production [1]. *Rhodotorula mucilaginosa* is a basidiomycetous red yeast capable of accumulating carotenoids and lipids and utilizing low-cost carbon sources [2,3,4]. Related red yeasts have also been developed as microbial hosts for heterologous terpene production, including the biosynthesis of several monoterpenes such as linalool [5,6,7]. Chen et al. engineered *R. mucilaginosa* JY1105 (CGMCC No. 2.6826) through *Agrobacterium*-mediated transformation (AMT) to produce α-terpineol, α-farnesene, and β-ionone, demonstrating that its endogenous isoprenoid pathway can support the biosynthesis of heterologous terpenoids [8]. However, that strategy relied on the random integration and expression of heterologous genes and did not demonstrate targeted gene disruption, thereby limiting precise and predictable strain engineering.

Genome editing could improve the precision of strain engineering, but CRISPR tools remain less developed in *R. mucilaginosa* than in model yeasts and several other non-conventional yeasts [9]. CRISPR/Cas9 systems have been established in the related red yeast *Rhodotorula toruloides* [10,11]. Notably, Jiao et al. developed a two-step workflow based on genome-integrated, codon-optimized *Staphylococcus aureus* Cas9 (SaCas9) followed by U6b-driven sgRNA delivery, generating indels at multiple loci and enabling donor-assisted homologous recombination [12]. A 2025 study also reported *TFR*- and *FPN1*-knockout strains of *R. mucilaginosa* in an investigation of iron-dependent carotenoid regulation [13].

SaCas9 is smaller than Streptococcus pyogenes Cas9 (SpCas9) and recognizes the longer 5′-NNGRRT-3′ protospacer-adjacent motif (PAM) [14,15]. SaCas9 and sgRNA had previously generated indels and enabled donor-assisted homologous recombination in the related red yeast *R. toruloides* [12]. These provided a documented framework for adaptation to *R. mucilaginosa* JY1105. In the present study, CrtYB was selected as a target gene with a readily observable phenotype. CrtYB encodes the bifunctional phytoene synthase/lycopene cyclase involved in carotenoid biosynthesis [16,17]. Disruption of this gene was therefore expected to reduce carotenoid accumulation and produce a depigmented colony phenotype suitable for preliminary screening, although sequence-level analysis remained necessary to confirm on-target editing. Accordingly, we developed an SaCas9-based editing workflow adapted to JY1105 and evaluated CrtYB disruption through phenotypic screening and sequence-level validation.

α-Bisabolol is a monocyclic sesquiterpene alcohol widely used in cosmetic and pharmaceutical formulations. It occurs naturally in the essential oils of *Matricaria recutita* and *Eremanthus erythropappus* [18,19,20]. Biosynthetically, α-bisabolol is formed from farnesyl diphosphate (FPP) through the catalytic activity of α-bisabolol synthase. Heterologous α-bisabolol production has been achieved in *Escherichia coli*, *Saccharomyces cerevisiae*, and *Komagataella phaffii* [21,22,23,24]. Through extensive pathway and fermentation engineering, including enhancement of the mevalonate pathway and FPP supply, attenuation of competing metabolic branches, and optimization of product recovery, some engineered strains have achieved gram-per-liter titers [21,22,24,25]. However, whether *R. mucilaginosa* can serve as a microbial chassis for α-bisabolol production has not yet been investigated.

In this study, we combined SaCas9-mediated genome editing with heterologous sesquiterpene production. A codon-optimized expression cassette containing the functionally characterized chamomile TPS1 sequence, which encodes α-bisabolol synthase [20], was introduced by random *Agrobacterium*-mediated integration into wild-type JY1105 and the depigmented R1-ΔCrtYB derivative. The resulting transformants were evaluated for α-bisabolol production by shake-flask fermentation and GC–MS analysis. To the best of our knowledge, this is the first report of heterologous α-bisabolol production using *R. mucilaginosa* as a microbial chassis. By coupling sequence-validated CrtYB editing with product formation, this study provides an SaCas9 workflow for JY1105 and provides a foundation for further metabolic engineering of *R. mucilaginosa*.

## 2. Materials and Methods

### 2.1. Strains and Media

*R. mucilaginosa* JY1105, isolated in our laboratory and deposited at the China General Microbiological Culture Collection Center (CGMCC 2.6826), served as the parental strain. Cultures were maintained on YPD solid medium (20 g/L glucose, 20 g/L peptone, 10 g/L yeast extract, 15 g/L agar) at 30 °C. When necessary, YPD plates were supplemented with bleomycin (50 μg/mL) or nourseothricin (50 μg/mL) and cefotaxime (100 μg/mL). *Escherichia coli* DH5α and *Agrobacterium tumefaciens* AGL1 were used for routine cloning and transformation, respectively, and were grown in LB medium (10 g/L NaCl, 10 g/L tryptone, 5 g/L yeast extract) at 37 °C or 30 °C, with kanamycin (50 μg/mL) as needed. Detailed strain information is provided in Table 1.

### 2.2. Plasmid Construction

The plasmids used in this study are listed in Table 1. A codon-optimized SaCas9 sequence (Table S1), derived from *Staphylococcus aureus*, was synthesized by SynBio Technologies (Suzhou, China). To construct pZPK-PGKp-Ble-NOSt-LDP1p-SaCas9-HSPt, SaCas9 was amplified from synthetic pUC57-SaCas9 using primers PGKp-fu-SaCas9-F and HSPt-fu-SaCas9-R and inserted into pZPK-PGKp-Ble-NOSt-LDP1p-HSPt via homologous recombination.

Target-specific sgRNA constructs were generated by restriction-free (RF) cloning [26]. Target fragments ΔCrtYB1–ΔCrtYB5 were amplified from synthetic pUC57-Amp-5stRNA-ΔCrtYB1-sgRNA using appropriate primer pairs to introduce homology arms. A second PCR using these fragments as megaprimers, using pUC57-Amp-5stRNA-ΔCrtYB1-sgRNA-Sup4 as the template, followed by DpnI digestion to remove the methylated template, generated the final sgRNA plasmids. These were subsequently combined with SaCas9 expression cassettes to obtain five final expression vectors: pZPK-5stRNA-ΔCrtYB1-sgRNA-Sup4-Ble-PGKp-SaCas9-HSPt through pZPK-5stRNA-ΔCrtYB5-sgRNA-Sup4-Ble-PGKp-SaCas9-HSPt. Primer sequences are provided in Table S2.

### 2.3. Western Blotting

Cells were harvested from 2 mL of overnight culture of each transformant in YPD medium, then the cells were disrupted using high-speed shaking at 4.0 m/s for 45 s, and the process was repeated 5 times with 3 min intervals of an ice bath between each round of shaking. The supernatants were recovered by centrifuging the suspensions at 12,000 g for 3 min. The total proteins were separated using SDS-PAGE with 12% polyacrylamide gels and transferred to the BioTrace™ NT nitrocellulose membrane (Pall, Beijing, China). The 6×His-tagged SaCas9 protein was detected using a mouse monoclonal anti-6×His antibody as the primary antibody and an HRP-conjugated goat anti-mouse IgG (H+L) antibody as the secondary antibody. Finally, a DAB horseradish peroxidase color development kit was used to visualize the blotting results [27].

### 2.4. sgRNA Design

sgRNAs were designed to target exonic regions near the 5′ end of *CrtYB* and to contain an SaCas9-compatible 5′-NNGRRT-3′ PAM. The 8–12 nt PAM-proximal seed region was required to be fully complementary to the target sequence [15]. Candidate protospacers were compared with the *R. mucilaginosa* JY1105 genome using BLASTn (https://blast.ncbi.nlm.nih.gov/Blast.cgi, accessed on 21 September 2026), and guides without predicted exact off-target matches were selected for plasmid construction.

### 2.5. Agrobacterium-Mediated Transformation and Mutant Verification

SaCas9-expressing isolates were first obtained by *Agrobacterium*-mediated transformation (AMT) using *A. tumefaciens* AGL1 carrying pZPK-PGKp-Ble-NOSt-LDP1p-SaCas9-HSPt. Each target-specific sgRNA construct was subsequently introduced into an SaCas9-expressing isolate by a second AMT step. Transformants were selected on YPD agar containing bleomycin (50 μg/mL) or nourseothricin (50 μg/mL) and cefotaxime (100 μg/mL) and were restreaked on fresh selective medium. Target loci were amplified by colony PCR with primers CrtYB-F and CrtYB-1000-R. Amplicons from candidate mutants were cloned and subjected to Sanger sequencing to define on-target alleles.

### 2.6. TPS1 Expression Cassette and Strain Construction

A synthetic *TPS1* coding sequence derived from *Matricaria recutita* sesquiterpene synthase 1 (GenBank KJ020282.1) [20] and codon-optimized for red yeast was amplified with EcoRV-TPS-F and SpeI-TPS-R (Table S2). The amplicon and pZPK expression vector were digested with EcoRV and SpeI and assembled as pZPK-PGKp-*TPS1*-P2A-Nat-HSPt. The construct was verified by colony PCR and sequencing in *E. coli* DH10B, transferred to *A. tumefaciens* AGL1, and introduced by AMT into JY1105 and R1-Δ*CrtYB* (a sequence-confirmed *CrtYB*-disrupted derivative of JY1105). For each genetic background, three independently obtained TPS1-positive transformants generated by random integration were randomly selected and confirmed by sequencing. Each transformant was evaluated in three biological replicate fermentations, and the highest-producing transformant was selected as the final engineered strain. The complete synthetic *TPS1* sequence is provided in Table S1.

### 2.7. Shake-Flask Fermentation and GC–MS Analysis

Each single transformed colony was inoculated into 20 mL YPD medium in a 100 mL shake flask and cultured at 30 °C and 200 rpm for 24 h to prepare the seed culture. A 5 mL aliquot of seed culture was then inoculated into 45 mL YTD50 medium (25 g/L tryptone, 10 g/L yeast extract, and 50 g/L glucose; initial pH 6.0) in a 250 mL shake flask and cultured at 30 °C and 200 rpm until the glucose in the fermentation medium was completely consumed. For extraction, 2 mL of fermentation broth was mixed with an equal volume of hexane and shaken at 200 rpm and 25 °C for 2 h. After centrifugation at 4000 rpm for 5 min, 500 μL of the hexane phase was transferred to a darkened glass vial. α-Bisabolol was analyzed using an Agilent 7890A gas chromatograph coupled to an Agilent 5975C inert XL EI/CI mass-selective detector equipped with a triple-axis detector (Agilent Technologies, Santa Clara, CA, USA). Separation was performed on a DB-WAX capillary column (30 m × 0.32 mm × 0.25 μm, Agilent Technologies). Helium was used as the carrier gas at a constant flow rate of 1.0 mL min^−1^. One microliter of the organic extract was injected at a split ratio of 10:1, and the inlet temperature was maintained at 250 °C. The oven temperature was initially held at 60 °C for 2 min, increased to 160 °C at 10 °C min^−1^, subsequently increased to 190 °C at 2 °C min^−1^, and finally increased to 240 °C at 5 °C min^−1^ and held for 10 min. The transfer-line, ion-source, and quadrupole temperatures were maintained at 250, 230, and 150 °C, respectively. Mass spectra were acquired in electron-ionization mode at 70 eV over an m/z range of 40–350. α-Bisabolol was identified by comparison of its retention time and EI mass spectrum with those of an authentic standard and by matching against the NIST mass spectral library. Quantification was performed using an external calibration curve prepared with authentic α-bisabolol. Each reported value for the selected strain therefore comprised three replicate fermentations, and results are presented as mean ± sample standard deviation.

## 3. Results

### 3.1. Construction and Expression of SaCas9 Strains

**Figure 1:**
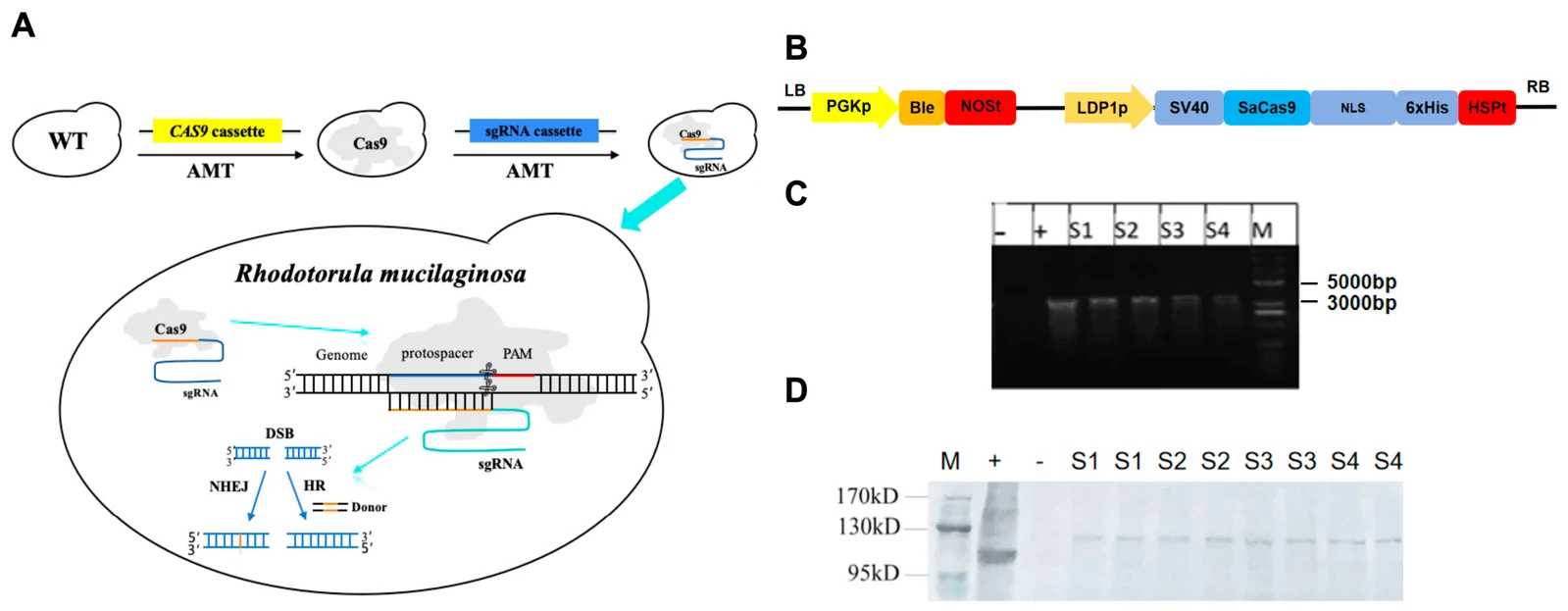
Construction and validation of the CRISPR/Cas9 system in *R. mucilaginosa* JY1105. (**A**) Workflow for system construction and schematic representation of sgRNA-guided SaCas9 cleavage. (**B**) SaCas9 expression cassette containing an N-terminal SV40 NLS and C-terminal nucleoplasmin NLS and a 6×His tag. (**C**) PCR analysis of independent *R. mucilaginosa* transformants. Lanes S1–S4, independent transformants; −, negative control; +, positive control; M, DNA marker. (**D**) Western blot analysis of SaCas9 expression. −, negative control; +, positive control; S1–S4, independent transformants; M, protein marker.

To establish the editing system in *R. mucilaginosa*, we generated SaCas9-expressing strains and subsequently introduced target-specific sgRNA constructs (Figure 1A). SaCas9 was fused to an SV40 nuclear-localization signal (NLS) at its N-terminus and to a nucleoplasmin NLS and 6×His tag at its C-terminus (Figure 1B). The SaCas9 expression vector was introduced into *A. tumefaciens* AGL1, and positive AGL1 transformants were used for AMT of *R. mucilaginosa* [8,28].

Bleomycin-resistant yeast transformants were obtained after AMT. PCR amplification of genomic DNA from four independent transformants produced the expected approximately 3.2 kb fragment (Figure 1C), consistent with integration of the SaCas9 cassette. Western blotting detected a 6×His-tagged protein at approximately 120 kDa in the transformed strains (Figure 1D), consistent with SaCas9 expression.

### 3.2. Targeted Mutagenesis of CrtYB

To evaluate the editing system, we targeted the endogenous *CrtYB* gene (Table S1), which encodes a bifunctional phytoene synthase/lycopene cyclase involved in carotenoid biosynthesis. Disruption of *CrtYB* was expected to produce a readily detectable reduction in colony pigmentation.

**Figure 2:**
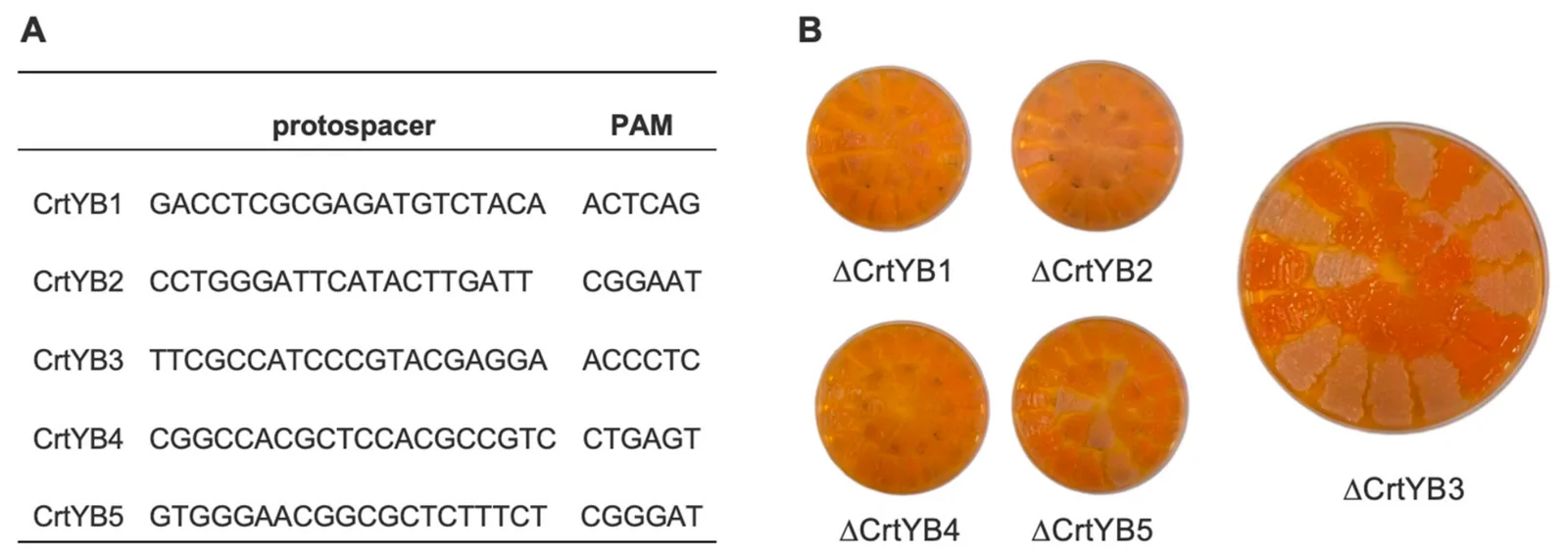
SaCas9-mediated editing of *CrtYB* in *R. mucilaginosa* JY1105. (**A**) Five 20 nt protospacers (CrtYB1–CrtYB5) and the corresponding SaCas9 PAM sequences. (**B**) Cultures recovered after transformation with the five target-specific sgRNA constructs.

Five 20 nt target sites near the 5′ end of the *CrtYB* coding region (CrtYB1–CrtYB5) were selected (Figure 2A). The corresponding guide sequences were incorporated into the sgRNA scaffold by RF cloning [26]. Three independent transformation experiments were performed for each of the five sgRNAs (CrtYB1–CrtYB5). For each sgRNA, 25 antibiotic-resistant colonies were randomly selected across the transformation batches for phenotype screening and Sanger sequencing of the target locus. *CrtYB3* and *CrtYB5* yielded 10/25 (40%) and 4/25 (16%) white colonies, respectively (Figure 2B). Genomic DNA was extracted from all 25 transformants, and the target region was amplified using the primers CrtYB-F and CrtYB-1000-R. All 10 white *CrtYB3* transformants contained 11–26 bp on-target deletions, and all 4 white *CrtYB5* transformants contained 18–26 bp on-target deletions (Figure S2). All red colonies retained the wild-type target sequence. *CrtYB1*, *CrtYB2*, and *CrtYB4* yielded no white colonies or target-site deletions. Thus, the observed sequence-validated editing frequencies were 40% for *CrtYB3*, 16% for *CrtYB5*, and almost 0% for the remaining guides under the documented screening conditions.

Four white-colony CrtYB3 transformants were randomly selected for sequence analysis. Genomic DNA was extracted from these transformants, and the target region was amplified using the primers CrtYB-F and CrtYB-1000-R. Sequence analysis showed that the four transformants carried deletions of 11, 14, or 26 bp adjacent to the sgRNA recognition sequence and PAM (Figure S2). These on-target deletions were consistent with repair through non-homologous end joining (NHEJ) following cleavage at the expected SaCas9 target site.

### 3.3. Heterologous TPS1 Construction and Product Identification

The codon-optimized TPS1 cassette was introduced independently into the JY1105 and R1-ΔCrtYB backgrounds by AMT. For each genetic background, three independently generated, TPS1-positive transformants carrying randomly integrated expression cassettes were selected for analysis. The presence of TPS1 in each selected transformant was verified by PCR amplification and sequence confirmation. Each transformant was subsequently cultured and processed independently for α-bisabolol extraction and analysis, with three fermentation-and-extraction replicates per transformant (*n* = 3).

**Figure 3:**
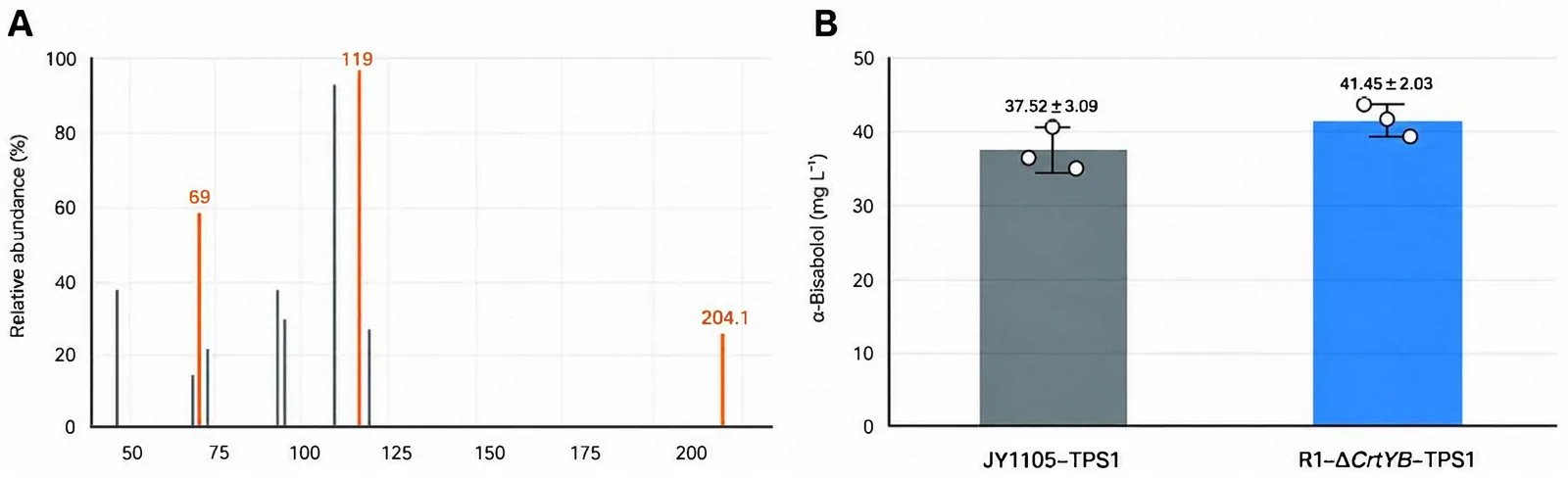
α-Bisabolol formation in selected transformants. (**A**) Representative EI mass spectrum of the approximately 28.72 min peak, with diagnostic ions at *m*/*z* 69, 119, and 204.(**B**) α-Bisabolol titers for the retained JY1105-TPS1 and R1-Δ*CrtYB*-TPS1 strains. Bars show mean ± SD.

Under the analytical conditions used, the wild-type JY1105 control lacking the TPS1 expression cassette showed no detectable chromatographic peak at the retention time of the authentic α-bisabolol standard. In contrast, representative transformants from the wild-type and edited backgrounds, designated JY1105–TPS1 and R1-ΔCrtYB–TPS1, respectively, exhibited peaks eluting at 28.64–28.77 min. These retention times were consistent with that of authentic α-bisabolol. Moreover, electron-ionization mass spectra of the corresponding peaks contained the characteristic fragment ions at *m*/*z* 69, 119, and 204, matching those observed for the authentic α-bisabolol standard (Figure 3A and Figure S1). Together, the retention-time and mass-spectral data support the assignment of these peaks as α-bisabolol and demonstrate that heterologous TPS1 expression enabled α-bisabolol formation in both the wild-type JY1105 and R1-ΔCrtYB backgrounds.

### 3.4. α-Bisabolol Production in Wild-Type and ΔCrtYB Backgrounds

Among the three independently obtained, random-integration *TPS1*-positive transformants evaluated for each genetic background, the clone showing the highest α-bisabolol titer was selected for subsequent characterization and designated as the final engineered strain. Following fermentation, the selected JY1105–TPS1 transformant produced 37.52 ± 3.09 mg/L α-bisabolol, whereas the selected R1-Δ*CrtYB*–TPS1 transformant produced 41.45 ± 2.08 mg/L (three fermentation-and-extraction replicates for each selected transformant, *n* = 3; Figure 3B and Table S4). Thus, α-bisabolol production was reproducibly detected in the selected engineered strains derived from both the wild-type JY1105 and R1-Δ*CrtYB* backgrounds.

The selected transformant in the R1-Δ*CrtYB* background showed a descriptively higher titer than that selected in the wild-type background (an absolute difference of 3.93 mg/L). However, this difference should not be interpreted as evidence that *CrtYB* disruption directly enhanced α-bisabolol production. Because *TPS1* was introduced by random genomic integration, the individual transformants may differ in integration locus, transgene copy number, cassette integrity, and local genomic context, any of which could influence *TPS1* expression and product formation. In addition, only the highest-producing clone among three transformants was selected from each background, rather than comparing matched integration events or a sufficiently large set of independently generated transformants. Therefore, the results establish production feasibility in both genetic backgrounds, while a causal effect of *CrtYB* disruption on α-bisabolol titer cannot be determined from the present data.

## 4. Discussion

Because *CrtYB* encodes the bifunctional phytoene synthase/lycopene cyclase required for carotenoid biosynthesis [16,17], its disruption generated a readily visible phenotype that facilitated preliminary screening. *CrtYB3* produced 10 white, deletion-containing colonies among 25 screened colonies (40%), and *CrtYB5* produced 4 such colonies among 25 (16%); all red colonies retained the wild-type target sequence. *CrtYB1*, *CrtYB2*, and *CrtYB4* yielded neither white colonies nor target-site deletions. Thus, the phenotype frequencies for *CrtYB3* and *CrtYB5* are fully supported by sequence-level validation of the corresponding screened colonies (Figure S2). Cas9-induced double-strand breaks can be repaired through non-homologous end joining (NHEJ) or homology-directed repair (HDR). Because no donor DNA template was supplied during *CrtYB* editing, the target-site deletions observed here are most consistent with error-prone NHEJ or related end-joining processes, which can generate small insertions or deletions and thereby disrupt the coding sequence. In contrast, HDR requires a homologous donor template and can enable precise sequence replacement or targeted integration. Donor-assisted repair was not assessed in the present study; therefore, we do not draw conclusions regarding HDR efficiency in JY1105. The relative contributions, efficiencies, and locus dependence of NHEJ and HDR in *R. mucilaginosa* JY1105 warrant systematic investigation in future work.

CRISPR/Cas9 tools have previously been developed in the related red yeast R. toruloides [10,11,12], demonstrating the feasibility of programmable genetic manipulation in oleaginous basidiomycetous yeasts. More recently, CRISPR/Cas9-mediated disruption of TFR and FPN1 in another *R. mucilaginosa* strain [13] further supports the applicability of targeted editing in this species. Nevertheless, efficient editing systems can remain species- and strain-dependent because transformation efficiency, DNA repair behavior, and expression of heterologous editing components may vary substantially among red yeasts.

In the present study, an SaCas9-based editing architecture was adapted to *R. mucilaginosa* JY1105. The successful disruption of *CrtYB* was confirmed by sequence-level validation, providing direct evidence that the platform can introduce the intended genomic modification in this strain. This work also builds on the earlier engineering of JY1105 through random AMT integration for the production of α-terpineol, α-farnesene, and β-ionone [8]. The novelty of the editing component is the adaptation of an SaCas9 workflow to *R. mucilaginosa* JY1105 and the sequence-validated disruption of *CrtYB* in this strain. This work extends prior CRISPR/Cas9 development in *R. toruloides* and the reported *TFR* and *FPN1* knockouts in another *R. mucilaginosa* context.

Accordingly, the contribution of this work is the adaptation of an SaCas9-based editing workflow to *R. mucilaginosa* JY1105 background and the sequence-validated disruption of *CrtYB* in this strain. This strain-specific editing capability expands the genetic toolbox available for *R. mucilaginosa* JY1105 and provides a foundation for future targeted pathway rewiring, functional-gene analysis, and construction of more predictable cell factories.

The previous JY1105 study demonstrated that the endogenous isoprenoid network of *R. mucilaginosa* can supply precursors for structurally distinct terpenoids [8]. Here, expression of the chamomile *TPS1* cassette, which encodes (−)-α-bisabolol synthase [20], extended the product spectrum of this host to a monocyclic sesquiterpene alcohol. Three random-integration *TPS1* transformants were evaluated per genetic background, and the highest-producing transformant from each background was selected. Both selected transformants produced α-bisabolol, with the R1-Δ*CrtYB*-derived transformant reaching 41.45 ± 2.08 mg /L. To the best of our knowledge, this is the first report of heterologous α-bisabolol production in *R. mucilaginosa*. The titer establishes feasibility but remains substantially below those achieved after extensive pathway and process engineering in established hosts, including 9.1 g /L in *E. coli*, 7.02 g/L in *S. cerevisiae*, and 32.8 g/L in *K. phaffii* [21,22,24]. These values are not directly comparable because the studies differed in host, fermentation scale, pathway engineering, extraction strategy, and process optimization.

The relatively modest shake-flask titer also identifies several routes for further development. Host-specific optimization could include stable targeted integration of *TPS1*, comparison of multiple independent isogenic transformants, controlled increases in gene copy number, and optimization of promoter strength and codon usage. Increasing MVA-pathway and FPP supply through enzymes such as tHMG1 and ERG20, while balancing the competing sterol, carotenoid, and lipid branches, has improved terpene production in yeast systems [8,21,22,24,25]. Because *R. mucilaginosa* naturally accumulates lipids and other hydrophobic metabolites [2,3,4], its lipid-rich physiology may also be exploited for intracellular product storage, although excessive suppression of lipid synthesis could compromise this advantage. Process strategies such as two-phase fermentation, improved oxygen transfer, and in situ product extraction may further increase α-bisabolol recovery. The SaCas9 workflow developed here provides a platform for testing these interventions through more controlled genome engineering.

Overall, the principal advance of this study lies in establishing a sequence-validated SaCas9-based genome-editing workflow for *R. mucilaginosa* JY1105. The targeted disruption of *CrtYB*, confirmed at the DNA-sequence level, demonstrates that the platform can generate defined genomic modifications in this strain rather than relying solely on random integration. In parallel, heterologous α-bisabolol biosynthesis was demonstrated in both the wild-type and edited JY1105 backgrounds. This product-oriented validation shows that *R. mucilaginosa* JY1105 can support heterologous sesquiterpene production while remaining amenable to targeted genetic modification. Together, these results provide a foundation for combining pathway introduction with rational genome editing to develop *R. mucilaginosa* JY1105 as a more controllable chassis for terpenoid biosynthesis.

## 5. Conclusions

An SaCas9-based genome-editing workflow was developed in *R. mucilaginosa* JY1105 and validated by sequence-confirmed deletions in CrtYB. Five guide RNAs targeting *CrtYB* were evaluated by sequencing 25 independently screened colonies per guide. Among the tested colonies, CrtYB3 produced sequence-confirmed edited clones at a frequency of 40% (10/25), whereas CrtYB5 yielded an editing frequency of 16% (4/25). No edited colonies were identified among the 25 colonies screened for each of CrtYB1, CrtYB2, and CrtYB4 under the conditions tested. These results demonstrate that the SaCas9 platform can mediate targeted genome modification in JY1105, while also showing substantial guide-dependent variation in editing performance.

To assess whether the JY1105 background and its edited derivative could support heterologous sesquiterpene biosynthesis, *TPS1* was subsequently introduced into both the wild-type JY1105 and R1-Δ*CrtYB* backgrounds. The selected JY1105–*TPS1* and R1-Δ*CrtYB*–*TPS1* transformants produced 37.52 ± 3.09 and 41.45 ± 2.08 mg/L α-bisabolol, respectively. These data establish α-bisabolol production in both genetic backgrounds. However, because *TPS1* was introduced by random integration and the highest-producing transformant was selected in each background, the observed difference in titer cannot be causally attributed to *CrtYB* disruption.

To the best of our knowledge, this is the first report of heterologous α-bisabolol production in *R. mucilaginosa*. Collectively, the work provides an SaCas9 editing tool for JY1105 and a proof of heterologous α-bisabolol production, supporting the further development of *R. mucilaginosa* as an editable chassis for targeted strain construction and heterologous sesquiterpene biosynthesis.

## Figures and Tables

**Table 1:** Strains and plasmids used in this study.

Strain or Plasmid	Relevant Characteristics	Source
**Strains**		
*Rhodotorula mucilaginosa* JY1105	MAT α	This lab
*Escherichia coli* DH5α	F^−^, φ80dlacZΔM15, Δ(lacZYA-argF)U169, deoR, recA1, endA1, hsdR17(rK^−^, mK^+^), phoA, supE44, λ^−^, thi-1, gyrA96, relA1	This lab
*Agrobacterium tumefaciens* AGL1	AGL0 recA::bla pTiBo542ΔT Mop^+^ CbR	This lab
R1-ΔCrtYB	JY1105 ΔCrtYB	This study
JY1105–TPS1	JY1105 TPS1 transformant	This study
R1-ΔCrtYB-TPS1	R1-ΔCrtYB TPS1 transformant	This study
**Plasmids**		
pUC57-Amp-5stRNA-ΔCrtYB1-sgRNA	Amp^R^	This study
pUC57-Amp-5stRNA-ΔCrtYB2-sgRNA	Amp^R^	This study
pUC57-Amp-5stRNA-ΔCrtYB3-sgRNA	Amp^R^	This study
pUC57-Amp-5stRNA-ΔCrtYB4-sgRNA	Amp^R^	This study
pUC57-Amp-5stRNA-ΔCrtYB5-sgRNA	Amp^R^	This study
pZPK-PGKp-Ble-NOSt-LDP1p-SaCas9-HSPt	Kan^R^	This study
pZPK-PGKp-TPS1-P2A-Nat-HSPt	Kan^R^	This study

## Data Availability

Target sequences and representative GC–MS outputs are provided in the Supplementary Materials. Original instrument reports and sequence files are available from the corresponding authors upon reasonable request.

## Supplementary Materials

The following supporting information can be downloaded at: https://www.mdpi.com/article/10.3390/microorganisms14102205/s1, Table S1. DNA sequences used in this study; Table S2. Primers used in this study; Table S3. SaCas9 target sequences used to edit CrtYB; Table S4. Replicate-level α-bisabolol titers for independently obtained TPS1-positive transformants; Figure S1. GC–MS comparison of an α-bisabolol standard and engineered-strain products; Figure S2. Sequence alignments of white CrtYB3 and CrtYB5 transformants.

## Abbreviations

The following abbreviations are used in this manuscript:

AMT	*Agrobacterium*-mediated transformation
NHEJ	Non-homologous end joining
NLS	Nuclear-localization signal
PAM	Protospacer-adjacent motif
RF	Restriction-free
sgRNA	Single-guide RNA
