# Supplementary material for: SaCas9-Mediated CrtYB Disruption Coupled with Heterologous α-Bisabolol Production in Rhodotorula mucilaginosa JY1105
Source: Microorganisms. 2026 Oct 1;14(10):2205. doi: 10.3390/microorganisms14102205 (PMC13707722; doi:10.3390/microorganisms14102205)
Supplement: Supplementary file 1 [file microorganisms-14-02205-s001.zip › microorganisms-4561907-supplementary.pdf]

Supplementary materials

SaCas9-Mediated *CrtYB* Disruption Coupled with Heterologous α-Bisabolol Production in *Rhodotorula mucilaginosa* JY1105

Wenyi Sun [1,†], Haoyang Liu [1,2,†], Yaqi Dong [2], Haoyu Li [2], Yanan Wang [2,*] and Qian Cai [1,*]

1 School of Pharmacy, Liaoning University of Traditional Chinese Medicine, Dalian 116600, China
2 State Key Laboratory Breeding Base of Dao-di Herbs, National Resource Center for Chinese Materia Medica, China Academy of Chinese Medical Sciences, Beijing 100700, China
† These authors contributed equally to this work.
* Correspondence: wangyn@nrc.ac.cn (Y.W.); caiqianmail@sina.com (Q.C.); Tel.: +86-138-1139-3185 (Y.W.); +86-159-9854-1866 (Q.C.)

Table S1. DNA sequences used in this study.

**Codon-optimized SaCas9 (3,258 bp)**<br>**3,258 bp; includes the terminal stop codon**
ATGGCGCCTAAGAAGAAGCGCAAGGTCGGCATTCACGGCGTCCCAGCCGCGAAGCGCAAC-<br>TACATCCTCGGCCTCGACATCGGCATCACCTCGGTCGGCTACGGCATCATCGACTACGAGACCCGCGACGTCATCGACGCCGGCGTCCG<br>CCTCTTCAAGGAGGCGAACGTCGAGAACAACGAGGGCCGCCGCTCGAA-<br>GCGCGGCGCGCGCCGCCTCAAGCGCCGCCGCCGCCACCGCATCCAGCGCGTCAAGAAGCTCCTCTTCGACTACAACCTCCTCACCGAC<br>CACTCGGAGCTCTCGGGCATCAACCCTTACGAGGCGCGCGTCAAGGGCCTCTCGCAGAA-<br>GCTCTCGGAGGAGGAGTTCTCGGCCGCGCTCCTCCACCTCGCGAAGCGCCGCGGCGTCCACAACGTCAACGAGGTCGAGGAGGACACC<br>GGCAACGAGCTCTCGACCAAGGAGCAGATCTCGCGCAACTCGAAGGCGCTCGAGGA-<br>GAAGTACGTCGCCGAGCTCCAGCTCGAGCGCCTCAAGAAGGACGGCGAGGTCCGCGGCTCGATCAACCGCTTCAAGACCTCGGACTAC<br>GTCAAGGAGGCGAAGCAGCTCCTCAAGGTCCAGAAGGCGTACCACCAGCTCGACCAG-<br>TCGTTCATCGACACCTACATCGACCTCCTCGAGACCCGCCGCACCTACTACGAGGGCCCAGGCGAGGGCTCGCCTTTCGGCTGGAAGGA<br>CATCAAGGAGTGGTACGAGATGCTCATGGGCCACTGCACCTACTTCCCAGAG-<br>GAGCTCCGCTCGGTCAAGTACGCGTACAACGCCGACCTCTACAACGCGCTCAACGACCTCAACAACCTCGTCATCACCCGCGACGAGA<br>ACGAGAAGCTCGAGTACTACGAGAAGTTCCAGATCATCGAGAACGTCTTCAAGCAGAAGAA-<br>GAAGCCGACCCTCAAGCAGATCGCGAAGGAGATCCTCGTCAACGAGGAGGACATCAAGGGCTACCGCGTCACCTCGACCGGCAAGCC<br>AGAGTTCACCAACCTCAAGGTCTACCACGACATCAAGGACATCACCGCGCGCAAGGA-<br>GATCATCGAGAACGCCGAGCTCCTCGACCAGATCGCGAAGATCCTCACCATCTACCAGTCGTCGGAGGACATCCAGGAGGAGCTCACC<br>AACCTCAACTCGGAGCTCACCCAGGAGGAGATCGAGCAGATCTCGAACCTCAAGGGCTACAC-<br>CGGCACCCACAACCTCTCGCTCAAGGCGATCAACCTCATCCTCGACGAGCTCTGGCACACCAACGACAACCAGATCGCGATCTTCAAC<br>CGCCTCAAGCTCGTCCCTAAGAAGGTCGACCTCTCGCAGCAGAAGGAGATCCCTAC-<br>CACCCTCGTCGACGACTTCATCCTCTCGCCAGTCGTCAAGCGCTCGTTCATCCAGTCGATCAAGGTCATCAACGCCATCATCAAGAAGT<br>ACGGCCTCCCTAACGACATCATCATCGAGCTCGCGCGCGAGAAGAACTCGAAGGAC-<br>GCCCAGAAGATGATCAACGAGATGCAGAAGCGCAACCGCCAGACCAACGAGCGCATCGAGGAGATCATCCGCACCACCGGCAAGGA<br>GAACGCCAAGTACCTCATCGAGAAGATCAAGCTCCACGACATGCAGGAGGGCAAGTGCCTC-<br>TACTCGCTCGAGGCGATCCCTCTCGAGGACCTCCTCAACAACCCGTTCAACTACGAGGTCGACCACATCATCCCTCGCTCGGTCTCGTTC<br>GACAACTCGTTCAACAACAAGGTCCTCGTCAAGCAGGAGGAGAACTCGAAGAAGGGCAACCG-<br>CACCCCTTTCCAGTACCTCTCGTCGTCGGACTCGAAGATCTCGTACGAGACCTTCAAGAAGCACATCCTCAACCTCGCGAAGGGCAAGG<br>GCCGCATCTCGAAGACCAAGAAGGAGTACCTCCTCGAGGAGCGCGACATCAAC-<br>CGCTTCTCGGTCCAGAAGGACTTCATCAACCGCAACCTCGTCGACACCCGCTACGCGACCCGCGGCCTCATGAACCTCCTCCGCTCGTA<br>CTTCCGCGTCAACAACCTCGACGTCAAGGTCAAGTCGATCAACGGCGGCTTCAC-<br>CTCGTTCCTCCGCCGCAAGTGGAAGTTCAAGAAGGAGCGCAACAAGGGCTACAAGCACCACGCCGAGGACGCGCTCATCATCGCGAA<br>CGCCGACTTCATCTTCAAGGAGTGGAAGAAGCTCGACAAGGCCAAGAAGGTCATGGAGAAC-<br>CAGATGTTCGAGGAGAAGCAGGCCGAGTCGATGCCAGAGATCGAGACCGAGCAGGAGTACAAGGAGATCTTCATCACCCCTCACCAG<br>ATCAAGCACATCAAGGACTTCAAGGACTACAAGTACTCGCACCGCGTCGACAAGAA-<br>GCCTAACCGCGAGCTCATCAACGACACCCTCTACTCGACCCGCAAGGACGACAAGGGCAACACCCTCATCGTCAACAACCTCAACGGC<br>CTCTACGACAAGGACAACGACAAGCTCAAGAAGCTCATCAACAAGTCGCCGGAGAA-<br>GCTCCTCATGTACCACCACGACCCTCAGACCTACCAGAAGCTCAAGCTCATCATGGAGCAGTACGGCGACGAGAAGAACCCTCTCTAC<br>AAGTACTACGAGGAGACCGGCAACTACCTCACCAAGTACTCGAAGAAGGACAAC-<br>GGCCCGGTCATCAAGAAGATCAAGTACTACGGCAACAAGCTCAACGCGCACCTCGACATCACCGACGACTACCCTAACTCGCGCAACA<br>AGGTCGTCAAGCTCTCGCTCAAGCCTTACCGCTTCGACGTCTACCTCGACAACGGCGTC-<br>TACAAGTTCGTCACCGTCAAGAACCTCGACGTCATCAAGAAGGAGAACTACTACGAGGTCAACTCCAAGTGCTACGAGGAGGCCAAG

AAGCTCAAGAAGATCTCGAACCAGGCCGAGTTCATCGCCTCGTTCTACAACAACGAC-
CTCATCAAGATCAACGGCGAGCTCTACCGCGTCATCGGCGTCAACAACGACCTCCTCAACCGCATCGAGGTCAACATGATCGACATCA
CCTACCGCGAGTACCTCGAGAACATGAACGACAAGCGCCCTCCTCGCATCATCAAGAC-
CATCGCGTCGAAGACCCAGTCGATCAAGAAGTACTCGACCGACATCCTCGGCAACCTCTACGAGGTCAAGTCGAAGAAGCACCCGCAG
ATCATCAAGAAGGGCAAGCGCCCAGCGGCGACCAAGAAGGCCGGCCAGGCGAAGAAGAAGAAGTAG

***CrtYB* sequence used for guide design and amplicon analysis (2,324 bp)**

ATGTGCTCGGCGCGTGCAGCCATCTACACTGGACGATCCCGCCTGCGGTCGTGCTTT-
GGAGCTTCTTCAGGAAACTCAGGACCTCGCGAGATGTCTACAAGACGTGCTTCTTGATCGTGGTAAGTCTCCGGTCCGGGATTCAGAGG
GCGCAGCAAACCCAGGTCCACTCATCCTGTGCCGCTCCCGCCACACAGATTGCAGTAACGG-
TACGGTTCACAACGCCTTCGGCTCCAAAGGCGTCAGCTGACCTTGGGACAAACCGGTGGGCGCACAGGCGACCATACCCTGGGATTCA
TACTTGATTCGGAATCGGGTGCGCAGCTCTCCAGCATATTGACCGCTGATCGTCAGA-
GAGCGAGGACTTGCTTACACAGGTCTCGGACCATCACGATCAGATCTGGTCCTATCCCGCCTCGTCCGTCGTGGGCCCGACCCTCTTCGC
CATCCCGTACGAGGAAGTCTTCTTCTTCTTCGTACAAACCTACATCACGGCCACGCTCCAC-
GCCGTCCTGAGTCGACCAGTCGTCCATGCCGTCCTACTCCCGACTAAAGCGAGCGAAGGCAGGCTCGCCCGACTCGTGGGAACGGCGC
TCTTTCTCGGGATCTTTGCGGTATCATGGGCGAAACTGGAAGAAGGCGGCGAGGGGAC-
GTATCTCGCGTTGATTGTCGGCTGGGTGGCACCGTTCTTGGCACTGCTCTGGTGAGCCTTGCTTCTCTTTGCCCCCCCCCCCCCCCTCTCTC
TCTCTCTCTCTCTCCGCGGGATACGTTTCCAGACGGGCAGAATTAAGCAGAAC-
CGACTTCCAGGTTTATCGCGTCGACGCACCTCATGGCGATGCCTTTGACGACCCTTTTGCCCGCCATCTTTGCGCCGACCCTCTACCTGTG
GGAGTGCGACGCCCGCGCGCTACAGCGTGGCACCTGGGTCATCGAGAAGGGTACCAA-
GCTCGGTTTGTCGTTCCGAGACCTCGAAATAGAGTGAGTGCATGGTGGTCGCTCGCCGTCGATGTACCGCAGCTGATTTCGTGCTATGAA
ATACTCGACAGGGAAGCAGTCTTTTTCCTGCTCACGAATGTGATGATCGTGTTCGG-
CATGGTCGCTTGGTGAGCAACTCCATCCGCTCGCACTCGCGTTAAATCTGCCATCTTGCAGCACTGTTTACTGACTCACATGATGTACAG
CGACTACTGCCTTGCCGTGCATGACCTTCGCTCATACGAAAA-
GCGGTCCTCGTCCGTCTTCCCGGCGGCGTCTGAGTTCCTCCCGATCCTGCTCTGGACACCCAAGCCTGCGGAGGCGGAGCGCATCAAGG
ATCTCCGAAACGCAATCGACATCCTTTCGGCACACTCCAAGAGCTTCTCGACTGCCAG-
CATGGTATTCGATGGCCGGTTGCGTCTCGACTTGCTCGCTTTGTACGTTCGCTCCGGTTCTCGTGCGCTGATCCTGGGCTAGGGCGCAACT
GTGCTCACACATGCGACGATTGCAGATACGCATGGTGCCGGGTGTGCGACGACCTGATCGAC-
GATGCATCGTCGGTGTCGACTGCAGAGGCAAACATTCGCCTCATCACCTCTTTCCTCGACCTCCTATACCCTCCGGGCAAGTCCAGCCCG
GACGCACATCCTCGTATCGTGCGCTCTACCGAGATCGACACCTTGTTGGCCGATCTCAAC-
GAGCCGCAGCGCGGATCGTTCCGCCTTCTCAGCCTGCTTCCGGTCACTCGTCCCCCTCTCGACGAGCTCGTCGCCGGCTTCCGCACGGAC
TTGTCCTTCCTCGCCAAAGCCTCTGGACGGAGCGGCGAGACGACCTCGAGCCCGACAAC-
GGCGACAGACCTCCCGATCGAGTCGGACGAGGATCTCCTCCTGTACGCCAACAACGTCGCGTCTTCGGTGGCGGACCTGTGCGTGCAGC
TCGTATGGGCGCATTCGGTTTCTTCATCACGACCGTCACGCCAAGAGCAGGAGGAAAC-
GCTCGCAGCGGCTCGCGAGATGGGCAAAGCATTGCAGCTCGTGAACATTGCTCGCGACGTCCCGGCCGACCTCAAGATCGGACGGATC
TACCTCCCCGGCCGATCGCTCGACGAACCAGTCGAGAACCTGACGGCAGACCGACGG-
GAGCTCCTTCGCCGTGCTCGCGGCATGGCGGACCGCAGCAAGGCGACGATCGAAAAGTTGCCGAGGGAGGCGCGCGGTGGGATCCGC
GCGGCGTGTTTGGTGTACTTGACGATCGGCGGGGCGGTCGACAAGGCGCTT-
GATGAAGGTCGCGTGCACGATAGAGCGGCGGTCAAGCGGGGGACGCGAGCGAGAACGGCCTGGGCTGCGCTCTGA

Codon-optimized *Matricaria recutita TPS1* (GenBank KJ020282.1; 1,719 bp)

ATGTCGACCCTCTCGGTCTCGACCCCCTCGTTCTCGTCGTCGCCCCTCTCGTCGGTCAACAA-
GAACTCGACCAAGCAGCACGTCACCCGCAACTCGGTCATCTTCCACGACTCGATCTGGGGCGACCAGTTCCTCGAGTACAAGGAGAAG
TTCAACGTCGCTACCGAGAAGCAGCTCATCGAGGAGCTCAAGGAGGAGGTCCGCAAC-
GAGCTCATGATCCGCGCCTGCAACGAGGCCTCGCGCTACATCAAGCTCATCCAGCTCATCGACGTCGTCGAGCGCCTCGGCCTCGCTTA
CCACTTCGAGAAGGAGATCGAGGAGTCGCTCCAGCACATCTACGTCACCTAC-
GGCCACAAGTGGACCAACTACAACAACATCGAGTCGCTCTCGCTCTGGTTCCGCCTCCTCCGCCAGAACGGCTTCAACGTCTCGTCGGA
CATCTTCGAGAACCACATCGACGAGAAGGGCAACTTCCAGGAGTCGCTCTGCAAC-
GACCCTCAGGGCATGCTCGCGCTCTACGAGGCCGCTTACATGCGCGTCGAGGGCGAGATCATCCTCGACAAGGCGCTCGAGTTCACCA
AGCTCCACCTCGGCATCATCTCGAACGACCCGTCGTGCGACTCGTCGCTCCGCACCGA-
GATCAAGCAGGCCCTCAAGCAGCCGCTCCGCCGCCGCCTCCCTCGCCTCGAGGCTGTCCGCTACATCGCCATCTACCAGCAGAAGGCTT
CGCACTCGGAGGTCCTCCTCAAGCTCGCCAAGCTCGACTTCAACGTCCTCCAGGA-
GATGCACAAGGACGAGCTCTCGCAGATCTGCAAGTGGTGGAAGGACCTCGACATCCGCAACAAGCTCCCTTACGTCCGCGACCGCCTC
ATCGAGGGCTACTTCTGGATCCTCGGCATCTACTTCGAGCCTCAGCACTCGCGCACCCG-
CATGTTCCTCATGAAGACCTGCATGTGGCTCATCGTCCTCGACGACACCTTCGACAACTACGGCACCTACGAGGAGCTCGAGATCTTCA
CCCAGGCTGTCGAGCGCTGGTCGATCACCTGCCTCGACGAGCTCCCCGAGTACATGAA-
GCTCATCTACCACGAGCAGTTCCGCGTCCACCAGGAGATGGAGGAGTCGCTCGAGAAGGAGGGCAAGGCGTACCAGATCCACTACATC
AAGGAGATGGCTAAGGAGGG-
CACCCGCTCGCTCCTCCTCGAGGCTAAGTGGCTCAAGGAGGGCTACATGCCTACCCTCGACGAGTACCTCTCGAACTCGCTCGTCACCT
GCGGCTACGCCCTCATGACCGCGCGCTCGTACGTCGCTCGCGACGACGGCATCGTCACCGAG-
GACGCTTTCAAGTGGGTCGCCACCCACCCCCCGATCGTCAAGGCTGCTTGCAAGATCCTCCGCCTCATGGACGACATCGCTACCCACAA
GGAGGAGCAGGAGCGCGGCCACATCGCCTCGTCGATCGAGTGCTACCGCAAGGAGAC-
CGGCGCCTCGGAGGAGGAGGCTTGCATGGACTTCCTCAAGCAGGTCGAGGACGGCTGGAAGGTCATCAACCAGGAGTCGCTCATGCCT

ACCGACGTCCCCTTCCCCCTCCTCATCCCGGCTATCAACCTCGCGCGCGTCTCG-GACACCCTCTACAAGGACAACGACGGCTACAACCACGCTGACAAGGAGGTCATCGGCTACATCAAGTCGCTCTTCGTCCACCCTATGATCGTCTGA

Table S2. Primers used in this study.

Primer name	Sequence (5' → 3')
PGKp-fu-SaCas9-F	CCTCCCCCGTGCAGCCCACCGATATCATGGCGCCTAAGAAGAAGCG
HSPt-fu-SaCas9-R	GTGAGACGGGGCGGAATCGTACTAGTCTAGTGATGGTGATGGTGGTG
pUC57-5stRNA-F	GTCACGACGTTGTAAAACGACGGCCAGTATCTGCGGCCATAC
sgRNA-ΔCrtYB2-5stRNA-R	CTGTTTCCAGAGTACTAAAACGACCTCGCGAGATGTCTACACGACGGTGAAGGGGATCGAAC
sgRNA-ΔCrtYB3-5stRNA-R	CTGTTTCCAGAGTACTAAAACAATCAAGTATGAATCCCAGGCGACGGTGAAGGGGATCGAAC
sgRNA-ΔCrtYB4-5stRNA-R	CTGTTTCCAGAGTACTAAAACGACGGCGTGGAGCGTGGCCGCGACGGTGAAGGGGATCGAAC
sgRNA-ΔCrtYB5-5stRNA-R	CTGTTTCCAGAGTACTAAAACAGAAAGAGCGCCGTTCCCACCGACGGTGAAGGGGATCGAAC
LB-fu-5stRNA-F	GCCGAATTGAATTCGAGCTCATCTGCGGCCATACCGCGATG
PGKp-fu-KpnI-SUP4-R	CTCAAGGTCCGTCTGGCCCGGGTACCAGACATAAAAAACAAAAAAATC
CrtYB-F	ATGTGCTCGGCGCGTGCAGCCATCTACAC
CrtYB-1000-R	GAAATCAGCTGCGGTACATCG
EcoRV-TPS-F	CGGGATATCATGGGCCGCAAGGAGTCG
SpeI-TPS-R	CCGACTAGTCTAGAGCTTCGCCTGCTCCC
TPS1020-R	CGCGTTGTGGAAGATGAAGCAG
PGK-F	CGCACTCGCTCTGGTACAGC

Table S3. SaCas9 target sequences used to edit *CrtYB*.

Guide	Protospacer (5′→3′)	PAM (5′→3′)
CrtYB1	GACCTCGCGAGATGTCTACA	ACTCAG
CrtYB2	CCTGGGATTCATACTTGATT	CGGAAT
CrtYB3	TTCGCCATCCCGTACGAGGA	ACCCTC
CrtYB4	CGGCCACGCTCCACGCCGTC	CTGAGT
CrtYB5	GTGGGAACGGCGCTCTTTCT	CGGGAT

Table S4. Replicate-level α-bisabolol titers for independently obtained TPS1-positive transformants.

Genetic background	Strain	Mean±SD（mg L^{-1}）
JY1105	JY1105–TPS1-1	32.86±3.24
JY1105	JY1105–TPS1-2	37.52±3.09
JY1105	JY1105–TPS1-3	30.26±2.62
R1-ΔCrtYB	R1-ΔCrtYB–TPS1-1	41.45±2.08
R1-ΔCrtYB	R1-ΔCrtYB–TPS1-2	37.20±2.85
R1-ΔCrtYB	R1-ΔCrtYB–TPS1-3	39.38±3.06

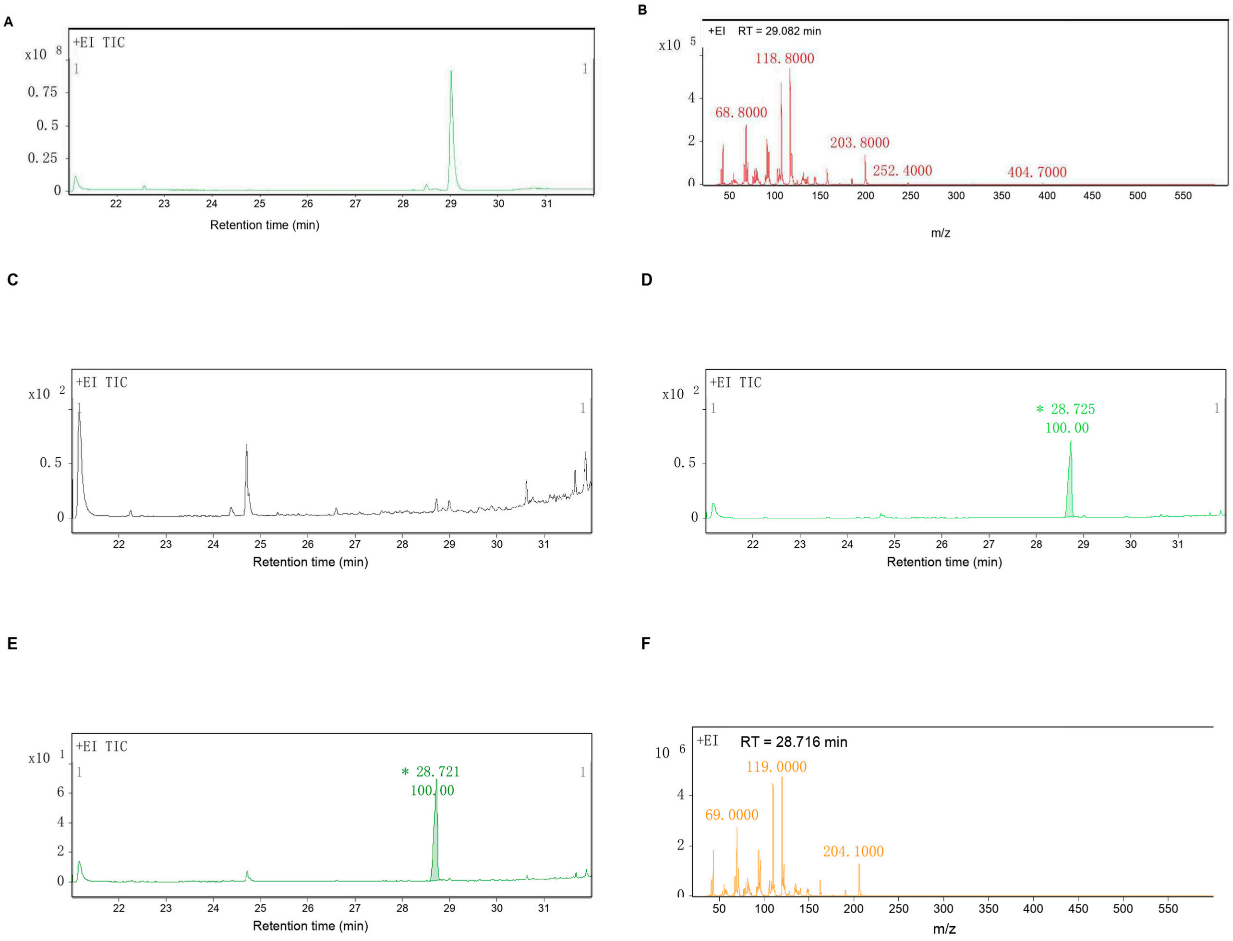

Figure S1. GC–MS comparison of an *α*-bisabolol standard and engineered-strain products. (A) Total-ion chromatogram of the standard. (B) EI mass spectrum of the 29.082-min standard peak. (C) JY1105 control. (D) JY1105–TPS1. (E) R1-ΔCrtYB–TPS1. (F) Representative EI mass spectrum of the 28.72-min peak in R1-ΔCrtYB–TPS1. The standard and sample spectra shared diagnostic ions at nominal *m*/*z* 69, 119, and 204.

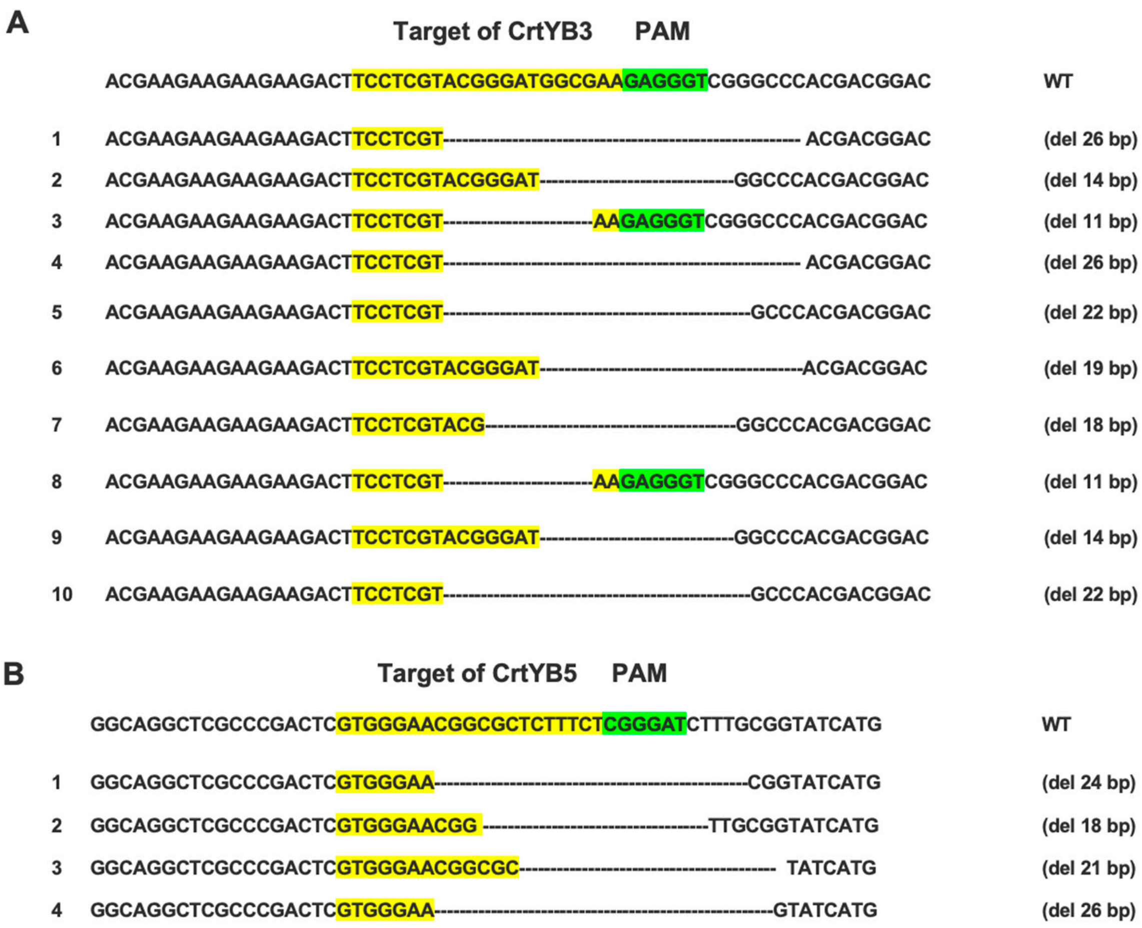

Figure S2. Sequence alignments of white CrtYB3 and CrtYB5 transformants.
(A) All 10 white CrtYB3 transformants carried 11–26-bp deletions. (B) All 4 white CrtYB5 transformants carried 18–26-bp deletions. Yellow shading indicates the protospacer and green shading indicates the PAM; dashes indicate deleted nucleotides. All red colonies retained the wild-type target sequence.
